# Palliative and prognostic approach in cancer patients identified in the multicentre NECesidades PALiativas 2 study in Argentina

**DOI:** 10.3332/ecancer.2021.1316

**Published:** 2021-11-10

**Authors:** Vilma Adriana Tripodoro, Victoria Llanos, María Laura Daud, Pilar Muñoz, Eden Del Mar, Romina Tranier, Sol Sandjian, Silvina De Lellis, Juan Manuel Días, Alvaro Saurí, Gustavo Gabriel De Simone, Xavier Gómez-Batiste

**Affiliations:** 1 Instituto de Investigaciones Médicas Alfredo Lanari, Universidad de Buenos Aires, Av Combatientes de Malvinas 3150, C1427 ARN, Buenos Aires, Argentina; 2 Instituto Pallium Latinoamérica, Bonpland 2257 (1425), Ciudad de Buenos Aires, Argentina; 3 Instituto de Oncología Ángel Roffo, Universidad de Buenos Aires, Av San Martín 5481 (1417), Ciudad de Buenos Aires, Argentina; 4 Hospital de Gastroenterología Dr. Carlos Bonorino Udaondo, Av Caseros 2061 (1264), Ciudad de Buenos Aires, Argentina; 5 Cátedra de Cuidados Paliativos, Universitat de Vic-Universitat Central de Catalunya, C Miquel Martí i Pol 1, 08500 Vic, Catalonia, Spain; a https://orcid.org/0000-0003-2328-6032

**Keywords:** advanced cancer, palliative care, chronic disease, prognosis, mortality

## Abstract

**Background:**

Early identification of palliative needs has proven benefits in quality of life, survival and decision-making. The NECesidades PALiativas (NECPAL) Centro Coordinador Organización Mundial de la Salud - Instituto Catalán de Oncología (CCOMS-ICO©) tool combines the physician’s insight with objective disease progression parameters and advanced chronic conditions. Some parameters have been independently associated with mortality risk in different populations. According to the concept of the ‘prognostic approach’ as a companion of the ‘palliative approach’, predictive models that identify individuals at high mortality risk are needed.

**Objective:**

We aimed to identify prognostic factors of mortality in cancer in our cultural context.

**Method:**

We assessed cancer patients with palliative needs until death using this validated predictive tool at three hospitals in Buenos Aires City. This multifactorial, quantitative and qualitative non-dichotomous assessment process combines subjective perception (the surprise question: Would you be surprised if this patient dies in the next year?) with other parameters, including the request (and need) for palliative care (PC), the assessment of disease severity, geriatric syndromes, psychosocial factors and comorbidities, as well as the use of healthcare resources.

**Results:**

2,104 cancer patients were identified, 681 were NECPAL+ (32.3%). During a 2-year follow-up period, 422 NECPAL+ patients died (61.9%). The mean overall survival was 8 months. A multivariate model was constructed with significant indicators in univariate analysis. The best predictors of mortality were: nutritional decline (*p* < 0.000), functional decline (*p* < 0.000), palliative performance scale (PPS) ≤ 50 (*p* < 0.000), persistent symptoms (*p* < 0.002), functional dependence (*p* < 0.000), poor treatment response (*p* < 0.000), primary cancer diagnosis (*p* = 0.024) and condition (in/outpatients) (*p* < 0.000). Only three variables remained as survival predictors: low response to treatment (*p* < 0.001), PPS ≤ 50 (*p* < 0.000) and condition (in/outpatients) (*p* < 0.000).

**Conclusion:**

This prospective model aimed to improve cancer survival prediction and timely PC referral in Argentinian hospitals.

## Introduction

Early identification of palliative care (PC) needs has proven benefits in quality of life, survival and decision-making [1]. However, the current PC model of as-needed referral and siloed departments can lead to heterogeneous access and fragmented care [2]. Furthermore, while integrated PC delivery gradually increases in the last year of life, disease-modifying and potentially curative care and unplanned care are not decreasing. Instead, it is rapidly increasing, particularly in the last 3 months of life [3]. Growing evidence shows that the NECesidades PALiativas (NECPAL) Centro Coordinador Organización Mundial de la Salud - Instituto Catalán de Oncología (CCOMS-ICO©) tool is validated with widespread use to identify patients likely in PC needs [4–8]. The combination of the surprise question (SQ) (Would you be surprised if this patient dies in the next year?) and some individual parameters may have potential prognostic utility for estimating mortality in patients with advanced chronic diseases (ACD), including cancer and non-cancer patients [9, 10]. Predictive models that identify individuals at high mortality risk are needed. Previous studies from our cultural context suggested that cancer patients might not have been detected promptly enough [6–8]. The NECPAL screening tool demonstrated feasibility in hospitals and primary care looking for the prevalence of unmet PC needs.

Recent findings adding a ‘prognostic approach’ enabling early PC intervention, multidimensional assessment, advanced care planning and case management for patients who may benefit from it [9]. The NECPAL prognostic model, including the SQ and some variables like functional decline, nutritional decline, multimorbidity (Charlson index), use of resources, disease-specific criteria of severity and progression plus age, generally performs well to predict 24-month mortality risk across different clinical conditions and care settings [9, 11].

In Argentina, only 10% of patients with PC needs have accessibility to PC services [12, 13]. In addition, the early palliative approach can prevent or ameliorate suffering and must be an informed option and available for all [2, 6, 14]. Therefore, we hypothesised that the timely identification supported by a predictive mortality risk model would be necessary for PC National Programme Design in Argentina [3, 15].

According to the concept of the ‘prognostic approach’ as an additional companion of the necessary ‘palliative approach’, we aimed to identify risk factors of mortality in cancer patients with PC needs in our cultural context [9]. Therefore, during a larger research project, ‘NECPAL 2 Study’, we identified and assessed cancer patients with PC needs until death at three hospitals in Buenos Aires City (Lanari and Roffo University of Buenos Aires Institutes and Udaondo Gastroenterology Hospital from the Government of Buenos Aires City). Additionally, 2 years after their identification, the survival analysis was collated with recent international findings [2, 9].

## Materials and method

As part of a more comprehensive intervention-based prospective exploratory design, all ≥ 18-year-old in/outcancer patients were identified and assessed in three hospitals as part of the NECPAL 2 Study (between June 2016 and July 2018). Following that, the individuals who met these criteria were evaluated regularly, as needed or at least once every 3months, looking for particular PC needs.

The data were gathered by interviews with the medical staff in charge according to the NECPAL CCOMS-ICO© tool V.3.0 [16]. Each hospital’s researchers were trained in NECPAL methodology following implementation procedures during a 1-month short training course [6]. Each patient’s interview lasted 10 minutes on average. This instrument offers a non-dichotomous multifactorial, quantitative and qualitative assessment method that incorporates subjective perception and the SQ: Would you be surprised if this patient dies in the next year? Positive answers (SQ+) meant that the doctor would not be surprised. Other specific indicators and the usage of healthcare resources were summarised in Table 1 [16]. Patients considered NECPAL positive (+) are those SQ+ patients who also fulfil at least one of the other indicators of the tool.

**Figure 1.**
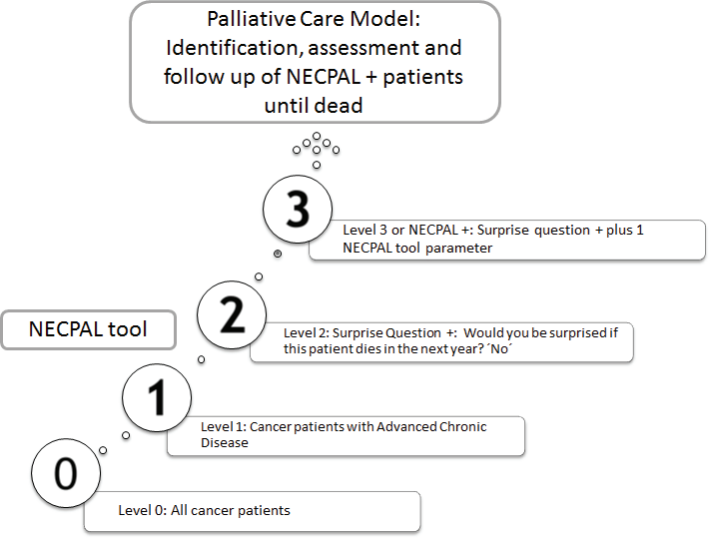
Levels of stratifications according to NECPAL methodology and the PCM. Adapted from Tripodoro *et al* [6].

**Figure 2.**
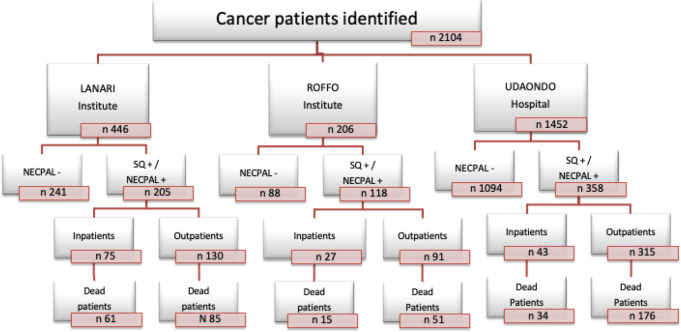
Recruitment process stratification. SQ, Surprise question; NECPAL+, Patients with SQ+ plus at least one of the other parameters of the tool; NECPAL−, Patients who his/her physician would be surprised if the patient would die during next year.

Cancer patients, identified only by their physicians in charge, were stratified into four levels according to NECPAL methodology (Figure 1) [6]. Level 0 diagnosed cancer; Level 1 cancer with ACD; Level 2 cancer with SQ+; Level 3 cancer with SQ+ and at least one indicator listed in Table 1 (NECPAL+). All NECPAL+ patients were followed for 2 years after first identification. A descriptive analysis was con ducted on the demographic and clinical indicators of these NECPAL+ patients. According to distribution, continuous variables were expressed as mean values ± standard deviation and median and range. The flowchart shows the recruitment process according to the institutions involved (Figure 2). It was sorted by levels, SQ+/NECPAL+, in/out and dead patients.

Survival curves of Kaplan–Meier were performed from the identification date of NECPAL+ patients until death or last control. A cut-off was made 2 years after detecting the first NECPAL+ patient (1 July 2018). All deceased patients were registered, and those referred to other centres or those lost to follow-up were censored. The median follow-up was established for the total sample and for those who had not died. Wald test and hazard ratio were used in Cox proportional analysis to compare groups according to age, gender, primary diagnosis, condition (in/outpatients), metastatic vital organ involvement, nutritional status and functional status (palliative performance scale (PPS) ≤ 50), or other indicators with statistical significance [17]. A multivariate model was constructed with the significant indicators in the univariate analysis with Cox proportional analysis to explore the best predictors of mortality. A value of *p* < 0.05 was considered significant. The statistical analysis was carried out by the Statistical Package for Social Science (IBM-SPSS 22 version (SPSS Inc. Chicago, IL)) and Stata V12.

The Ethics Committees approved this study of the institutions involved in the NECPAL 2 Study. It was registered at the National Ministry of Health (RENIS IS001867/IS001871) and funded by the Argentina National Cancer Institute and Pallium Latinoamérica.

## Results

**Figure 3.**
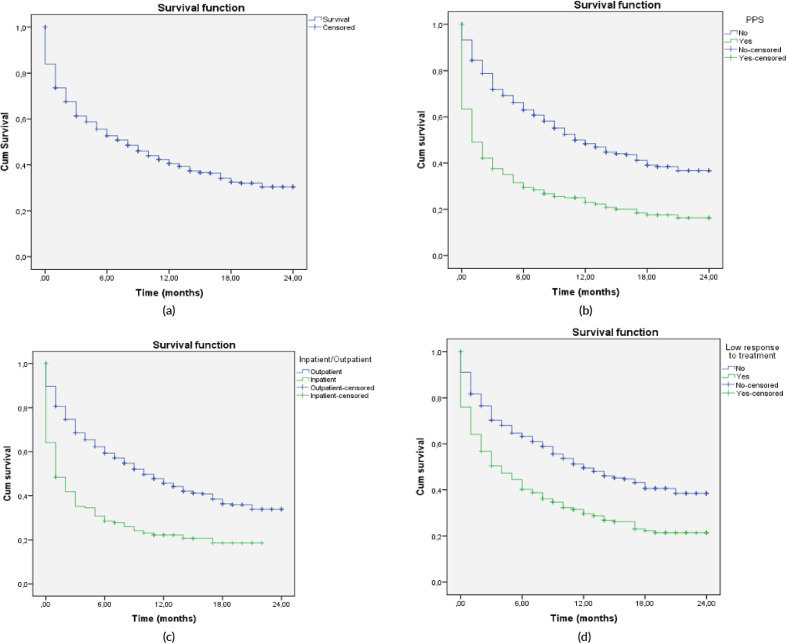
Kaplan–Meier global survival curves and significant predictors of mortality (n = 681). (a): NECPAL+ patients global survival. (b): Survival in NECPAL+ patients and significant functional decline, PPS ≤ 50. (c): Survival in NECPAL+ in/outpatients. (d): Survival in NECPAL+ patients with low response to treatment.

A total of 2,104 cancer patients were found, with 681 being NECPAL+ (32.3%). During the 2-year follow-up period after identification, 422 NECPAL+ patients died (61.9%) (Figure 2). The data were collated from three hospitals. Table 2 summarises sociodemographic results, primary cancer diagnoses and NECPAL indicators. A multivariate model was constructed where variables with a higher/lower probability of survival were: nutritional decline (*p* 0.000), functional decline (*p* 0.000), PPS ≤ 50 (*p* 0.000), persistent symptoms (*p* 0.002), functional dependence (*p* 0.000), low response to treatment (*p* 0.000), primary cancer diagnosis (*p* 0.024) and condition (in/outpatients) (*p* 0.000). The only three variables that remained as predictors were: low response to treatment (*p* 0.001), PPS ≤ 50 (*p* 0.000) and condition (in/outpatients) (*p* 0.000). Table 3 characterises the multivariate model. The mean overall survival was 8 months. Figure 3 shows global survival curves (Figure 3a) and significant predictors of mortality from 681 NECPAL+ patients (Figure 3b–d).

## Discussion

The breakthrough of this multicentric study was the use of a direct prospective method aimed to improve cancer survival prediction and PC referral in hospital settings in Argentina. The overall survival was 8 months, which confirms that we identified patients in their last year of life or less. Furthermore, it enabled us to design a palliative care model (PCM) as part of a more extensive study and a prognosis model approach. This model has the potential to be replicated in other hospitals throughout Argentina [6].

The NECPAL tool was feasible and helpful in our multicentric setting to identify mortality risk factors coinciding with Spanish results [14, 18]. The 12 months’ time frame is exactly the NECPAL tool’s point of interest. After 12- and 24-month follow-up, the survival rate endorses the conceptual approach of typical trajectories of decline in ACD, including cancer [4, 10, 19, 20]. In Scotland, while PC is increasing, curative care is growing even faster in the last months of life. Therefore, identifying populations with limited prognoses is central to enhancing policymaking, service planning and delivery [9, 21].

NECPAL’s predictive validity had already been tested in a Spanish cohort of a prevalence study, with high sensitivity and negative predictive value but limited specificity in predicting 24-month death [22]. Later, some NECPAL parameters have been independently associated with mortality risk in different populations [23]. In a previous study, we constructed a predictive model in one of the current Argentinian hospitals to identify individuals with PC needs for 24 months [8]. In that project, also focused on cancer patients, almost half of NECPAL+ patients had a significant functional decline. The concurrence of all such indicators correlated strongly with high mortality risk during the first month of follow-up. Half of the patients in that population died within 4 months suggests that the referral to PC services might have been too late. In the current study, the mean survival was two times the previous one. This outcome opened a new question about the potential influence of the systematic approach and the reflexive SQ on earlier referral to PC. The research team was involved with staff daily practice, both oncologists and PC teams. We considered this influence as positive, and it might be a catalytic agent looking for an earlier palliative approach.

Turrillas *et al* [9] pointed out that a prognostic tool using NECPAL variables selected based on clinicians’ expertise and literary evidence can be accurate and helpful in predicting 2-year mortality in patients with advanced conditions [9]. Nutritional and functional decline, severe and refractory dyspnoea, multimorbidity, use of resources and specific disease indicators were potentially prognostic variables for mortality across four clinical groups and end-of-life trajectories: cancer, dementia and neurologic diseases, chronic organ failure and frailty [23].

A progressive increase in the mortality risk was observed based on the number of clinical factors in the NECPAL model ([9]. All six parameters were combined into three prognostic groups regardless of the indicator (0–2, 3–4 and 5–6 parameters). This model showed accuracy in predicting survival in oncological patients who were the illest. The prevalence of four out of six predictive variables was higher than in the other groups (non-cancer patients), including a high presence of the disease-specific criterion of severity, which could explain a better performance [9].

We have included cancer patients exclusively in the project. Frailty, which is strongly associated with survival rate, has been included as a specific criterion in the latest version of the NECPAL tool [9]. We did not assess frailty by using specific tools. However, this should be a future research field in our population.

There has been a significant increase in the use of PC services by oncologists. However, PC patients’ referral occurs late in the trajectory of illness, at an average of 30–60 days before death. In addition, most families who refer to PC programmes state that they preferred an earlier consultation [24].

Hui *et al* [25] compared the quality of care between early (>3 months between first PC consultation and death) and late (≤3 months) PC referral in cancer patients [25]. This cut-off was chosen because the median time from referral to death to the outpatient clinic was approximately 3 months. In addition, a PC referral is often triggered by symptom distress rather than prognosis [2]. However, the ‘prognosis approach’ should, from a pragmatic point of view, estimate prognosis together with PC needs, supporting oncologists in their daily practice at the clinical and organisational levels.

In the current study, the NECPAL tool identified these patients with PC needs and confirmed their mortality risk in 1 year. In addition, this systematic screening allowed us to assess patients’ multidimensional needs by a multidisciplinary PC team in a mean of the last 8 months of their life (95% CI: 6.3–9.7) [26].

It is estimated that 69%–82% of cancer deaths would benefit from PC, improving quality of life, patient and caregiver distress and even survival [27]. As a result, multiple calls have been for integrating PC into standard oncology care to achieve the best outcomes [28–31]. Therefore, we admitted these patients to our PCM, collaborating with the oncologist and PC team, providing periodic assessment until death [26].

Despite the growing evidence supporting the effectiveness of palliative outpatient clinics, only 59% of the National Cancer Institute-designated cancer centres reported having such clinics in the USA [24, 32]. Moreover, even with a PC clinic available, only 46% of patients had their first PC visit in the outpatient setting at MD Anderson Cancer Center, Houston, Texas [2]. In contrast, we assessed 79% of outpatients with advanced cancer and PC needs. It suggests that our PCM aimed to integrate PC into cancer follow-up. However, these findings should not be generalisable to other contexts because we probably did not reach the whole cancer outpatients sample.

### Limitations and strengths

These results are directly applicable to the institutions involved, and it is not generalisable to other hospitals. One of the main strengths of this analysis is the involvement of three hospitals (two university hospitals, one of them oncological and a gastroenterological diseases referent hospital), allowing us to evaluate the PCM quality across medical conditions and care settings. Selection bias should be considered because identifying at-risk individuals with PC needs was based on clinical judgment. In addition, a substantial number of patients were classified as having functional or nutritional decline based on a subjective decision and not necessarily relying on validated criteria.

## Conclusions

We propose that developing the ‘palliative and prognosis approach’ will respond to an urgent need to investigate the most effective but time-efficient method to assess patients’ multidimensional needs with cancer and deliver timely PC. Despite the uncertainty in survival prediction, this model should facilitate clinical decision-making by providing an approximate timeframe (1 year or months). Future research should focus on validating new prognostic factors and linking them to decision-making. According to our earlier studies, the screening approach proved practical and accessible on a broader scale [6–8]. We want to make sure that everyone has access to the best cancer care possible. In addition to strengthening the timely palliative approach in national hospitals, the National Cancer Institute’s National PC Programme could consider this methodology in future policymaking [15].

## Author contributions

All the authors have made substantial intellectual contributions to this article. For example, VT, VLL, MLD, PM, AS, GDS and XGB contributed to study design; VT, VLL, MLD, RT, EDM and SS in the data collection; VT, VLL, SDL and JMD in analysis and interpretation of data; VT, VLL and SDL in the writing of the manuscript and all authors in the decision to submit the manuscript for publication and revised the final version.

## Conflicts of interest

The authors declare that they have no conflicts of interest.

## List of abbreviations

PC, Palliative care; ACD, Advanced chronic disease; PCM, Palliative care model; SQ, Surprise question; PPS, Palliative performance scale.

## Figures and Tables

**Table 1.** The NECPAL CCOMS-ICO© tool: general indicators of severity and progression and disease-specific indicators [8, 16].

The NECPAL tool indicators	
Choice, request or need of palliative approach	Has the patient or the main caregiver requested palliative/comfort treatments exclusively or suggests limitation of therapeutic effort? Do healthcare professionals consider that the patient requires PC or palliative treatment at this moment?
Functional markers	Serious established functional dependence (Barthel score < 20) Loss of two or more ADLs even though there is adequate therapeutic intervention or clinical perception of functional decline (sustained, intense/severe, progressive, irreversible) not related to concurrent conditions
Nutritional markers	Serum albumin <2.5 g/dl, not related to acute episodes of unbalance Weight loss >10% or clinical perception of nutritional decline (sustained, intense/severe, progressive, irreversible) not related to concurrent conditions
Emotional	Presence of emotional distress with psychological symptoms (sustained, intense/severe, progressive) not related to acute concurrent conditions
Geriatric syndromes in the last 6 months	Persistent pressure ulcers (stages III–IV), recurrent infections (>1), delirium, persistent dysphagia, falls (>2)
Comorbidity	*Charlson Index* [11]
Additional factors on use of resources	Two or more urgent (unplanned) hospital (or skilled nursing facilities) admissions due to chronic disease in the last year. Need of complex/intense continuing care, either at an institution or at home
Cancer (1 single criterion)	Confirmed diagnosis of metastatic cancer who present low response or contraindication of specific treatment, progressive outbreak during treatment or metastatic affectation of vital organs Significant functional deterioration (PPS ≤ 50%) [17] Persistent, troublesome symptoms, despite optimal treatment of underlying condition(s)
Chronic pulmonary disease (≥2 criteria)	Breathlessness at rest or on minimal exertion between exacerbations. Difficult physical or psychological symptoms despite optimal tolerated therapy. FEV1 < 30% or criteria of restricted severe deficit: FVC < 40%/DLCO < 40%. Accomplishment of oxygen therapy at home criteria. Recurrent hospital admissions (>3 admissions in 12 months due to exacerbations)
Chronic heart disease (≥2 criteria)	Heart failure NYHA stage III or IV, severe valve disease or inoperable coronary artery disease. Shortness of breath at rest or minimal exertion. Difficult physical or psychological symptoms despite optimal tolerated therapy. Ejection fraction severely affected (<30%) or severe pulmonary hypertension (>60 mm Hg). Renal failure (GFR < 30 L/minute). Repeated hospital admissions with symptoms of heart failure/ischaemic heart disease (>3 in the last year)
Serious chronic liver disease (1 single criterion)	Advanced cirrhosis: stage Child C, MELD-Na Score > 30 or with one or more of the following medical complications: diuretic-resistant ascites, hepatorenal syndrome or upper gastrointestinal bleeding due to portal hypertension with failed response to treatment. Hepatocellular carcinoma: present, in stage C or D (BCLC)
Serious chronic renal disease (1 single criterion)	Serious renal failures (GFR < 15) in patients to whom substitutive treatment or transplant is contraindicated
Chronic neurological diseases [1]: CVA (1 single criterion)	During acute and subacute phases (<3 months post stroke): persistent vegetative or minimal conscious state >3 days. During the chronic phase (>3 months post stroke): repeated medical complications (aspiration pneumonia, pyelonephritis, recurrent febrile episodes, pressure ulcers stages 3–4 or dementia with severe criteria post stroke)
Chronic neurological diseases [2]: MND, multiple sclerosis and Parkinson (≥2 criteria)	Progressive deterioration in physical and/or cognitive function despite optimal therapy. Complex and difficult symptoms. Speech problems with increasing difficulty communicating. Progressive dysphagia Recurrent aspiration pneumonia, breathless or respiratory failure
Dementia (≥2 of the following criteria)	Severity criteria: GDS/FAST 6c or more. Progression criteria: loss of two or more ADLs in the last 6 months, despite adequate therapeutic intervention or difficulty swallowing, or denial to eat, in patients who will not receive enteral or parenteral nutrition. Use of resources criteria: multiple admissions (>3 in 12 months, due to concurrent processes – aspiration pneumonia, pyelonephritis, sepsis, etc. – that cause functional and/or cognitive decline)

ADL, Activities of daily living; BCLC, Barcelona clinic liver cancer; CVA, Cerebrovascular accident; DLCO, Diffusing capacity of the lung for carbon monoxide; FEV1, Forced expiratory volume in 1 s; FVC, Forced vital capacity; GFR, Glomerular filtration rate; NYHA, New York Heart Association; MND, Motor neuron disease; GDS/FAST, Global deterioration scale/functional assessment; MELD-Na, Model for end-stage liver disease

**Table 2.** NECPAL+ patient distribution by characteristics and hospital.

	Total NECPAL+ (*n* = 681)	Lanari Institute (*n* = 205)		Roffo Institute (*n* = 118)		Udaondo Hospital (*n* = 358)	
		Inpatient (*n* = 75)	Outpatient (*n* = 130)	Inpatient (*n* = 27)	Outpatient (*n* = 91)	Inpatient (*n* = 43)	Outpatient (*n* = 315)
Female	339 (50%)	35 (47%)	72 (56%)	9 (33%)	53 (59%)	19 (44%)	150 (48%)
Age (mean)	65 (23–99)	76 (50–93)	76 (38–99)	56 (27–77)	63 (33–81)	60 (32–99)	59 (23–91)
Nutritional decline	259 (38%)	42 (56%)	37 (29%)	10 (37%)	19 (21%)	23 (53%)	128 (41%)
Functional decline	266 (39%)	54 (72%)	66 (51%)	18 (67%)	33 (37%)	17 (40%)	76 (24%)
Functional dependence	89 (13%)	24 (32%)	17 (13%)	8 (30%)	5 (6%)	7 (16%)	27 (9%)
Breast cancer	36 (5%)	7 (9%)	24 (19%)	4 (15%)	1 (1%)	0 (0%)	0 (0%)
Lung cancer	118 (17%)	8 (11%)	23 (18%)	3 (11%)	83 (92%)	0 (0%)	1 (0%)
Gastrointestinal cancer	413 (61%)	31 (41%)	25 (19%)	7 (26%)	1 (1%)	43 (100%)	305 (97%)
Genitourinary cancer	35 (5%)	8 (11%)	22(17%)	2 (7%)	3 (3%)	0 (0%)	0 (0%)
Oncohaematologic	11 (2%)	5 (7%)	6 (5%)	0 (0%)	0 (0%)	0 (0%)	0 (0%)
Gynaecologic cancer	12 (2%)	1 (1%)	7 (5%)	4 (15%)	0 (0%)	0 (0%)	0 (0%)
Other	40 (6%)	9 (12%)	19 (15%)	7 (26%)	2 (2%)	0 (0%)	3 (1%)
Primary Unknown	16 (2%)	6 (8%)	3 (2%)	0 (0%)	0 (0%)	0 (0%)	6 (2%)

**Table 3.** Multivariate model.

	Exposed	Not exposed	Hazard ratio (95% CI)	p value	Wald test
PPS ≤ 50^a^	205	474	1.699 (1.351–2.137)	< 0.000	20.582
Inpatient^a^	145	534	1.682 (1.327–2.132)	< 0.000	18.496
Low response to treatment^b^	291	380	1.409 (1.143–1.738)	< 0.001	10.323

a 2 missing cases

b 10 missing cases
